# Relationship between uric acid levels and risk of chronic kidney disease in a retrospective cohort of Brazilian workers

**DOI:** 10.1590/1414-431X20176048

**Published:** 2017-08-07

**Authors:** L.S.N. Chini, L.I.S. Assis, J.R. Lugon

**Affiliations:** 1 Divisão de Medicina Interna, Departamento de Medicina, Universidade Federal Fluminense, Niterói, RJ, Brasil; 2 Universidade Federal do Estado do Rio de Janeiro, Rio de Janeiro, RJ, Brasil; 3 Divisão de Nefrologia, Departamento de Medicina, Universidade Federal Fluminense, Niterói, RJ, Brasil

**Keywords:** Chronic kidney disease, Hyperuricemia, Glomerular filtration rate

## Abstract

Uric acid (UA) levels are increased in patients with kidney dysfunction. We analyzed the association between asymptomatic hyperuricemia and new-onset chronic kidney disease (CKD). A retrospective cohort study was designed to collect data from employees of an energy generation and distribution company in the city of Rio de Janeiro, Brazil, who had undergone the company’s annual medical checkup from 2008 to 2014. People with ≤2 years of follow-up, with baseline estimated glomerular filtration rate (eGFR) <60 mL·min^-1^·(1.73 m^2^)^-1^ or with incomplete data were excluded. The endpoint was defined as eGFR <60 mL·min^-1^·(1.73 m^2^)^-1^ estimated through the chronic kidney disease epidemiology collaboration equation (CKD-EPI). The study included 1094 participants. The mean follow-up period was 5.05±1.05 years and 44 participants exhibited new-onset CKD. The prevalence of hyperuricemia was 4.2%. There was a significant inverse correlation between baseline serum levels of UA and baseline eGFR (R=-0.21, P<0.001). Female gender (OR=4.00; 95%CI=1.92-8.29, P<0.001) and age (OR=1.06; 95%CI=1.02-1.11, P=0.004) but not UA levels (OR=1.12; 95%CI=0.83-1.50; P=0.465) were associated with new-onset CKD. Diabetes mellitus and body mass index were independent factors for fast progression (OR=2.17; 95%CI=1.24-3.80, P=0.007 and OR=1.04; 95%CI=1.01-1.07; P=0.020). These results did not support UA as an independent predictor for CKD progression in the studied population.

## Introduction

Chronic kidney disease (CKD) is a worldwide public health problem and its management, by either dialysis or transplantation, imposes a considerable economic burden to health systems (1). Its prevalence is high and continues to increase, especially in developing countries (2). Studies have suggested that early diagnosis and treatment of modifiable CKD risk factors are important to prevent the progression to renal replacement therapy (3,4). Established predictors for development of CKD include high blood pressure and diabetes mellitus (5,6).

Uric acid (UA) is the end product of purine metabolism in humans, and its high serum levels, hyperuricemia, are classically related to the precipitation of crystals in the joints, leading to arthritis (7). There is increasing evidence of the association of hyperuricemia with hypertension and cardiovascular disease (8,9). However, whether hyperuricemia is a predictor of CKD or only a consequence of reduced UA excretion is still not clear (10).

In recent years, a large number of observational studies have examined the potential relationship between hyperuricemia and development of CKD. These studies have shown conflicting results. Feig examined 12 epidemiology studies, finding a positive correlation in 8 and absence of association in 4 (11). Two meta-analysis published in 2014 favored the relationship when the follow-up period was long enough or when participants were middle-aged (12,13). However, to date, this subject is still a matter of controversy (14). Therefore, the aim of the present study was to analyze the association between asymptomatic hyperuricemia and new-onset CKD in employees of an energy generation and distribution company in the city of Rio de Janeiro, Brazil.

## Material and Methods

### Study population

This was a retrospective cohort study. Data was collected from employees of an energy generation and distribution company in the city of Rio de Janeiro, Brazil, who had undergone the company’s annual medical checkup from 2008 to 2014. The study population was composed of office workers in non-laborious activities during work hours. To certify that only cases of asymptomatic hyperuricemia were studied, participants with a history of gout attacks were excluded. Those with ≤2 years of follow-up, with estimated glomerular filtration rate (eGFR) <60 mL·min^–1^·(1.73 m^2^)^–1^ in the first exam or with incomplete data were also excluded.

Sociodemographic information, physical examination data and biochemical analysis data were based on the participant’s first year medical examination. A second result of serum creatinine was obtained from the last annual checkup.

### Disease definition

Hyperuricemia was defined as a UA of >6.0 mg/dL in women and >7.0 mg/dL in men (15). CKD was defined as an eGFR <60 mL·min^–1^·(1.73 m^2^)^–1^ (10). The eGFR was estimated using the chronic kidney disease epidemiology collaboration equation (CKD-EPI) (16), as follows:

If female and serum creatinine (sCr) ≤0.7 mg/dL: eGFR=144 × (sCr/0.7)^–0.329^×0.993^age^;

If female and sCr >0.7 mg/dL: eGFR=144 × (sCr/0.7)^–1.209^×0.993^age^;

If male and sCr ≤0.9 mg/dL: eGFR=141 × (sCr/0.9)^–0.411^×0.993^age^;

If male and sCr >0.9 mg/dL: eGFR=141 × (sCr/0.9)^–1.209^×0.993^age^.

Hypertension was defined as systolic blood pressure of ≥140 mmHg, diastolic blood pressure ≥90 mmHg or current use of antihypertensive medication, according to the Seventh Report of the Joint National Committee on the Prevention, Detection, Evaluation, and Treatment of High Blood Pressure (17). The diagnosis of diabetes mellitus was defined as a fasting blood glucose level of at least 126 mg/dL, or current use of glucose-lowering agents (18). Metabolic syndrome and its components were diagnosed employing the harmonizing definition. This required the presence of at least three of five parameters: increase waist circumference (≥90 cm in men and ≥80 cm in women), triglyceride ≥150 mg/dL or treatment for hypertriglyceridemia, high density lipoprotein cholesterol (HDL-C) <40 mg/dL for men and <50 mg/dL for women or drug treatment for this abnormality, systolic blood pressure ≥130 mmHg or diastolic blood pressure ≥85 mmHg or treatment for high blood pressure and fasting glucose ≥100 mg/dL or treatment for high glucose (19).

Blood pressure was measured in the left arm in the sitting position, after 5 min at rest, using an aneroid sphygmomanometer with an accuracy of 5 mmHg. The first and fifth Korotkoff sounds were recorded, and the mean value of 3 measurements was used for analysis. Body weight was measured with light clothing and without shoes by an adjustable scale (Filizola, Brazil). Height was measured without shoes using a stadiometer to the nearest 0.1 cm. The body mass index (BMI) was calculated as the weight (in kilograms) divided by the square of the height (in meters). All measurements were taken by trained nurses.

Higher education was defined as a complete post-secondary education. Sedentary lifestyle was defined as doing irregular or no physical activity.

The biochemical analyses were performed in samples obtained after at least 12 h of fasting. The serum levels of fasting plasma glucose, creatinine, total cholesterol, HDL-C, and triglycerides were measured by enzymology using the automated chemistry analyzer Selectra-E (Wiener Lab, Argentina) or the Labimax 240 premium (Hirose Electronic System CO., Ltd., Japan). Serum UA was measured by an enzymatic method (Liquiform, Labtest, Brazil) with intra- and total variation coefficients of 0.82–0.97 and 1.14–1.48%, respectively.

### Statistical analysis

Statistical analyses were conducted using the Statistical Package for Social Sciences version 18.0 (SPSS Inc., USA). The results of continuous variables are reported as mean and standard deviation if distribution was Gaussian, and alternatively as median and range if the distribution was not normal. Categorical variables are reported as frequencies. Comparisons between two groups were carried out by the unpaired *t-*test in case of normal distribution or, alternatively, by its nonparametric equivalent, the Mann-Whitney test. Pearson or Spearman correlation tests were used as appropriate. Logistic regression was used to test for association of independent variables with eGFR <60 mL·min^–1^·(1.73 m^2^)^–1^. P values less than 0.05 were considered significant.

### Ethical approval

The study was conducted with the approval of the Research Ethical Committee of the Faculdade de Medicina, Universidade Federal Fluminense, Niterói, RJ, Brazil (CAAE: 44061515.7.0000.5243).

## Results

**Figure 1.**
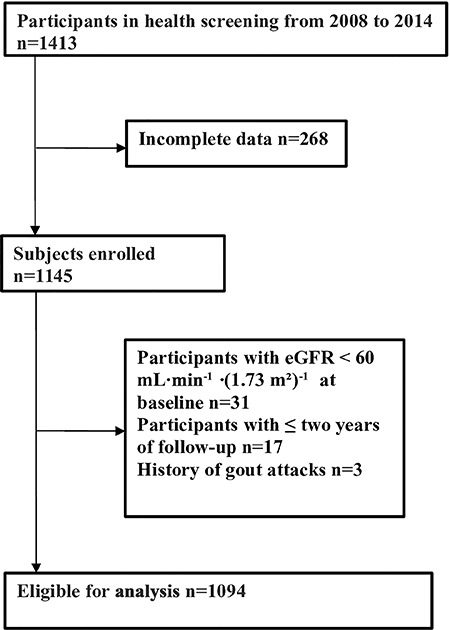
Selection of study participants.

From a total of 1413 participants, 1094 were selected for the final analysis (Figure 1). Of those excluded due to an insufficient follow-up, none developed eGFR below 60 mL·min^–1^·(1.73 m^2^)^–1^. The mean follow-up period of the study population was 5.05±1.05 years. Their baseline characteristics are shown in Table 1. The mean age of patients was 48.7±8.8 years. The participants were predominantly male and white, 12% were current smokers, and 4.2% had hyperuricemia. Sixty patients (5.5%) were diabetic, with 33 diagnosed with diabetes mellitus by the start of the study and undergoing treatment. From those under treatment, 16 (48%) had a fast blood glucose <126 mg/dL. There were 313 hypertensive patients (28.6%), 153 (49%) of them under treatment, from which 86 (56.2%) had controlled blood pressure. Of note, 47.3% of the sample had metabolic syndrome. Mean BMI was statistically higher in hyperuricemic participants and so did the systolic and diastolic blood pressure, serum levels of triglycerides, total cholesterol and creatinine (for creatinine the difference was restricted to males). As a consequence, the prevalence of metabolic syndrome in hyperuricemic patients was also higher. There was a weak but significant inverse correlation between baseline serum levels of UA and baseline eGFR (R=-0.21, P<0.001).

**Table 1.** Baseline characteristics of participants.

	All	HU- (95.8%)	HU+ (4.2%)	P value
n	1094	1048	46	-
Male gender (%)	822 (75.0%)	787 (75.1%)	35 (76.1%)	0.879
Age (years)	48.7±8.8	48.7±8.9	49.1±8.4	0.774
White race (%)	999 (91.3%)	958 (91.4%)	41 (89.1%)	0.751
Higher education (%)	659 (60.2%)	633 (60.4%)	26 (56.5%)	<0.001
Sedentary lifestyle (%)	577 (52.2%)	541 (51.6%)	29 (63.0%)	0.129
Current smoker (%)	131 (12%)	128 (12.2%)	3 (6.5%)	0.244
BMI (kg/m^2^)	27.1±4.6	27.1±4.5	29.9±5.5	<0.001
Systolic BP (mmHg)	119.4±16.5	119.1±16.4	126.6±18.2	0.002
Diastolic BP (mmHg)	76.2±11.0	76.0±11.0	81.6±10.9	0.001
Fasting plasma glucose (mg/dL)	99.0±21.7	98.8±21.9	103.5±17.9	0.154
Triglycerides (mg/dL)	135.4±85.3	132.2±83.6	209.7±92.3	<0.001
Total cholesterol (mg/dL)	202.0±37.2	201.5±37.2	214.6±37.6	0.019
HDL-C (mg/dL)	43.1±11.0	43.3±11.0	39.5±11.5	0.023
Creatinine (mg/dL)				
Male	0.79±0.16	0.78±0.16	0.84±0.16	0.036
Female	0.62±0.17	0.61±0.17	0.69±0.16	0.114
eGFR, mL·min^-1^·(1.73 m^2^)^-1^	105.6±14.8	105.8±14.9	100.2±14.1	0.013
Uric acid (mg/dL)				
Male	4.9±1.2	4.8±1.0	7.7±0.6	<0.001
Female	3.6±1.2	3.5±1.0	6.8±0.7	<0.001
Metabolic syndrome (%)	517 (47.3%)	477 (45.5%)	40 (87%)	<0.001
Diabetes mellitus (%)	60 (5.5%)	56 (5.3%)	4 (8.7%)	0.328
Hypertension (%)	313 (28.6%)	294 (28.1%)	19 (41.3%)	0.052

Data are reported as means±SD, or number and %. HU: hyperuricemia; BP: blood pressure; BMI: body mass index; HDL-C: high-density lipoprotein cholesterol; eGFR: estimated glomerular filtration rate using CKD-EPI equation. Statistical analysis was done with the *t*-test and chi-square test or their non-parametric equivalent, as appropriate.

The eGFR varied along the study from 105.6±14.8 to 87.9±17.8 mL·min^–1^·(1.73 m^2^)^–1^ (P<0.001) resulting in a mean decline of 17.7±18.8 mL·min^–1^·(1.73 m^2^)^–1^. The mean annual rate of progression was 3.5±4.3 mL·min^–1^·(1.73 m^2^)^–1^. Patients were classified according to the rate of progression, following a previous study (3). In 19.7% of cases no progression was observed; in 4.7% the annual rate of progression was between zero to 1 mL·min^–1^·(1.73 m^2^)^–1^, characterizing a mild progression; in 30.0%, the progression was moderate [>1 to 4 mL·min^–1^·(1.73 m^2^)^–1^]; and in 45.6%, fast [>4 mL·min^–1^·(1.73 m^2^)^–1^]. Serum levels of uric acid of each group were 4.8±1.3, 4.6±1.3, 4.5±1.3, and 4.5±1.3 mg/dL, respectively, without statistically significant differences between groups. At the end of follow-up, 44 cases (4%) exhibited eGFR <60 mL·min^–1^·(1.73 m^2^)^–1^.

To assess the association of selected parameters with the development of eGFR <60 mL·min^–1^·(1.73 m^2^)^–1^, we resorted to a multivariate logistic regression model (Table 2). Serum UA level, the variable of primary interest, was not found to be associated with the dependent variable. In fact, only female gender (OR=4.00; 95%CI=1.92–8.29; P<0.001) and age, (OR=1.06; 95%CI=1.02–1.11; P=0.004) were statistically associated with the development of CKD. Serum HDL-C levels tended to be protective but statistical significance was not reached at the final analysis (OR=0.97; 95%CI=0.94–1.00; P=0.079).

To better understand the risk factors for progression of CKD in the study population, a second model of multivariate logistic regression was run using fast progression as the dependent variable (Table 3). Here, diabetes mellitus and BMI were found as independent factors for fast progression (OR=2.17; 95%CI=1.24–3.80, P=0.007, and OR=1.04; 95%CI=1.01–1.07; P=0.020, respectively).

**Table 2.** Multivariate logistic regression model for predictors of development of glomerular filtration rate <60 mL·min^-1^·(1.73 m^2^)^-1^ at the end of follow-up.

Predictive variables	OR	95%CI	P
Uric acid (mg/dL)	1.12	0.83–1.50	0.465
Female gender	4.00	1.92–8.29	0.001
Age (years)	1.06	1.02–1.11	0.004
Diabetes mellitus	0.79	0.18–3.55	0.762
Hypertension	0.99	0.47–2.08	0.980
HDL-C (mg/dL)	0.97	0.94–1.00	0.079
Triglycerides (mg/dL)	1.00	0.99–1.00	0.332
BMI (kg/m^2^)	0.98	0.90–1.06	0.550
Sedentary lifestyle	0.95	0.51–1.79	0.884
Smoking	0.93	0.37–2.29	0.870

OR: odds ratio; CI: confidence interval; HDL-C: high-density lipoprotein cholesterol; BMI: body mass index.

**Table 3.** Multivariate logistic regression model for predictors of fast progression along the follow-up.

Predictive variables	OR	95% CI	P
Uric acid (mg/dL)	0.90	0.81–1.01	0.084
Female gender	0.84	0.61–1.17	0.305
Age (years)	1.01	0.99–1.02	0.403
Diabetes mellitus	2.17	1.24–3.80	0.007
Hypertension	0.77	0.58–1.03	0.076
HDL-C (mg/dL)	1.00	0.99–1.01	0.766
Triglycerides (mg/dL)	1.00	0.99–1.00	0.981
BMI (kg/m^2^)	1.04	1.01–1.07	0.020
Sedentary lifestyle	0.92	0.72–1.17	0.493
Smoking	0.76	0.52–1.12	0.160

OR: odds ratio; CI: confidence interval; HDL-C: high-density lipoprotein cholesterol; BMI: body mass index.

## Discussion

In this retrospective cohort study of Brazilian office workers, we investigated the association between asymptomatic hyperuricemia and new-onset CKD. At baseline, serum UA levels exhibited a weak but significant inverse correlation with eGFR. Along the study, 4 percent of the participants developed new-onset CKD, but asymptomatic hyperuricemia was not statistically associated with such endpoint. Indeed, in the multivariate analysis, the only factors associated with development of CKD were age and female gender, a finding consistent with a recent systematic review and meta-analysis addressing this subject (20). It should be commented that we did not find association of diabetes and hypertension with new-onset CKD. An absence of association of diabetes and new-onset CKD had already been reported in two retrospective cohorts (21,22). Characteristics of the study design could in part explain this apparent discrepancy. As participants with baseline eGFR <60 mL·min^–1^·(1.73 m^2^)^–1^ were excluded from the analysis, the follow-up period may not have been long enough to detect the development of CKD in patients with a high baseline eGFR. This subject was additionally explored in the second multivariate logistic regression model in which diabetes was confirmed as a strong independent factor associated with a fast progression rate. It should also be pointed out that an adequate control of diabetes and hypertension is well recognized as a factor that attenuates progression to CKD (10). In this regard, a large proportion of our hypertensive and diabetic patients was undergoing treatment and exhibited controlled blood pressure and glycemic status.

Despite several years of epidemiological and clinical research, the real significance of hyperuricemia as a predictor of CKD progression remains obscure. A number of studies have reported that UA is an independent risk factor for the development of kidney disease, while others have reported negative results (23–25). In a large retrospective cohort study of 94,422 Taiwanese participants, Wang et al. (26) demonstrated an independent link between hyperuricemia and incident CKD after 3.5 years of follow-up. Kawashima et al., in a retrospective cohort study of 1285 Japanese male workers, also found that asymptomatic hyperuricemia was a predictor of new-onset CKD after 18 years of follow-up (21). On the other hand, Chonchol et al. (27) in a cohort study of 5808 participants found that UA had a strong cross-sectional association with prevalent CKD and was a weak independent predictor of progression of kidney dysfunction. Yen et al. described similar results in a cohort study of 800 elderly Taiwanese subjects during a period of 2 years (28).

Some authors studied the relationship of hyperuricemia and long-term outcomes in patients with CKD. Madero et al. evaluated 838 patients with CKD stage 3 to 4 and found that hyperuricemia appeared to be an independent risk factor to all-cause mortality and cardiovascular mortality, but not kidney failure (29). In a recent study, Nacak et al. (30) concluded that UA is not associated with decline in renal function in a referred cohort of 2466 patients with stage 3, 4, and 5 CKD in Sweden.

Randomized controlled trials (RCT) evaluating the effects of UA lowering therapy on renal outcomes were reviewed in two meta-analysis. Zhang et al. reviewed seven RCT involving 451 cases and identified that UA lowering therapy could delay the progression of CKD (31). Otherwise, a meta-analysis of eight RCT evaluating 476 participants concluded that there was insufficient evidence to currently recommend widespread use of UA lowering therapy to slow the progression of CKD (32).

There are a number of mechanisms by which high levels of UA increases the risk for CKD development. In a study in rats, hyperuricemia increased systemic blood pressure, proteinuria, renal dysfunction, progressive renal scarring and induced vascular disease via a COX-2-dependent pathway (33). Another study in hyperuricemic rats fed with a low-salt diet demonstrated glomerular hypertension, which appeared to be due to insufficient vasoconstriction of the afferent arteriole (34). There is clinical evidence that hyperuricemia is associated with hypertension due to endothelial dysfunction (35).

The understanding of the biological function of UA is far from elucidated. UA is recognized as a powerful antioxidant and is responsible for approximately half of the antioxidant capacity of human plasma. On the other hand, the molecule can also function as a pro-oxidant, either by generating free radicals during its degradation or by stimulating NADPH oxidase (36). Hyperuricemia results from interaction of several risk factors such as nutrition, social-economic status, gender, age, genetic and environment, making its prevalence variable in different populations and areas. In the present study, which included a healthy, young and well-educated population, the prevalence of hyperuricemia was extremely low, affecting only 4.2% of the subjects, in contrast to 13.2% reported by Rodrigues et al. in a Brazilian population (37). The prevalence is higher in developed countries perhaps as a consequence of diet, sedentary lifestyle and an increase in obesity and hypertension, reaching 21.4% among US adults (38). In China, due to rapid economic changes, the prevalence is increasing and a meta-analysis found a rate of 13.3% (39). We could not find a definite explanation for the controversial findings about the role of UA as a predictor of new-onset CKD between studies.

Our study involved a relatively large sample. Participants had uniform access to health services and ease of follow-up, since the annual medical checkup was standardized. For legal reasons, medical records and laboratory results need to be kept for 20 years allowing precise monitoring. Nevertheless, some limitations must be considered. First, a single baseline measurement of UA was used to predict events several years later. However, a number of previous studies have resorted to the same approach. Second, the subjects were limited to employees of one company in Rio de Janeiro, predominantly composed of whites and not representative of the general Brazilian population. Third, the definition of renal progression was based on changes in eGFR, rather than more precise measurements of renal function, such as proteinuria or iothalamate clearance. Finally, the prevalence of hyperuricemia was very low in the cohort.

In conclusion, our findings indicate that UA had a weak, but significant, cross-sectional association with baseline eGFR and was not correlated with new-onset CKD in a specific group of Brazilian workers. These results did not support UA as an independent predictor for CKD progression in the studied population. The question of whether a study addressing poorer segments of the Brazilian population would lead to a similar conclusion should be investigated.
